# mastR: an R package for automated identification of tissue-specific gene signatures in multi-group differential expression analysis

**DOI:** 10.1093/bioinformatics/btaf114

**Published:** 2025-03-17

**Authors:** Jinjin Chen, Ahmed Mohamed, Dharmesh D Bhuva, Melissa J Davis, Chin Wee Tan

**Affiliations:** Bioinformatics Division, Walter and Eliza Hall Institute of Medical Research, Melbourne, VIC 3052, Australia; Department of Medical Biology, Faculty of Medicine, Dentistry and Health Sciences, University of Melbourne, Parkville, VIC 3010, Australia; Bioinformatics Division, Walter and Eliza Hall Institute of Medical Research, Melbourne, VIC 3052, Australia; Department of Medical Biology, Faculty of Medicine, Dentistry and Health Sciences, University of Melbourne, Parkville, VIC 3010, Australia; Bioinformatics Division, Walter and Eliza Hall Institute of Medical Research, Melbourne, VIC 3052, Australia; Department of Medical Biology, Faculty of Medicine, Dentistry and Health Sciences, University of Melbourne, Parkville, VIC 3010, Australia; South Australian immunoGENomics Cancer Institute (SAiGENCI), Faculty of Health and Medical Sciences, The University of Adelaide, Adelaide, SA 5005, Australia; Bioinformatics Division, Walter and Eliza Hall Institute of Medical Research, Melbourne, VIC 3052, Australia; Department of Medical Biology, Faculty of Medicine, Dentistry and Health Sciences, University of Melbourne, Parkville, VIC 3010, Australia; South Australian immunoGENomics Cancer Institute (SAiGENCI), Faculty of Health and Medical Sciences, The University of Adelaide, Adelaide, SA 5005, Australia; Frazer Institute, Faculty of Medicine, The University of Queensland, Brisbane, QLD 4102, Australia; Department of Clinical Pathology, Faculty of Medicine, Dentistry and Health Sciences, University of Melbourne, Parkville, VIC 3010, Australia; Bioinformatics Division, Walter and Eliza Hall Institute of Medical Research, Melbourne, VIC 3052, Australia; Department of Medical Biology, Faculty of Medicine, Dentistry and Health Sciences, University of Melbourne, Parkville, VIC 3010, Australia; Frazer Institute, Faculty of Medicine, The University of Queensland, Brisbane, QLD 4102, Australia

## Abstract

**Motivation:**

Biomarker discovery is important and offers insight into potential underlying mechanisms of disease. While existing biomarker identification methods primarily focus on single cell RNA sequencing (scRNA-seq) data, there remains a need for automated methods designed for labeled bulk RNA-seq data from sorted cell populations or experiments. Current methods require curation of results or statistical thresholds and may not account for tissue background expression. Here we bridge these limitations with an automated marker identification method for labeled bulk RNA-seq data that explicitly considers background expressions.

**Results:**

We developed *mastR*, a novel tool for accurate marker identification using transcriptomic data. It leverages robust statistical pipelines like *edgeR* and *limma* to perform pairwise comparisons between groups, and aggregates results using rank-product-based permutation test. A signal-to-noise ratio approach is implemented to minimize background signals. We assessed the performance of *mastR*-derived NK cell signatures against published curated signatures and found that the *mastR*-derived signature performs as well, if not better than the published signatures. We further demonstrated the utility of *mastR* on simulated scRNA-seq data and in comparison with *Seurat* in terms of marker selection performance.

**Availability and implementation:**

*mastR* is freely available from https://bioconductor.org/packages/release/bioc/html/mastR.html. A vignette and guide are available at https://davislaboratory.github.io/mastR. All statistical analyses were carried out using R (version ≥4.3.0) and Bioconductor (version ≥3.17).

## 1 Introduction

Biomarkers are biological features that infer the states of cells, tissues, or individuals, either diseased or healthy. Biomarkers may include molecular features like genes, and proteins which can be used in research and clinical settings to provide insights into disease diagnosis, prognosis, and treatment. In recent years, biomarkers have been identified through various -omics approaches, including transcriptomics, proteomics, and metabolomics, providing an improved overview of the molecular landscape of the system being studied (Lawlor *et al.* 2009, Wang and Yu 2013, Rodrigues *et al.* 2016). This influx of omics data has advanced the development of computational and bioinformatics methods to identify biomarkers, providing opportunities to accelerate biomarker discovery and thereby facilitating diagnostic and therapeutic developments for various diseases and cancers (Kaur *et al.* 2021, Lee and Kim 2021, Vlachavas *et al.* 2021). However, separating the background signal of the tissue microenvironment from the target marker’s signal remains a complex problem.

While many recent marker identification methods focus on emerging data types such as single-cell RNA sequencing (scRNA-seq) or spatial omics data, where disease signals are more easily separated from background, bulk RNA-seq remains a valuable resource due to its widespread availability, cost and already established pool of datasets. Existing tools for bulk RNA-seq marker identification, such as *edgeR* (Robinson *et al.* 2010), *limma* (Ritchie *et al.* 2015), or *DESeq2* (Love *et al.* 2014), require manual curation of differential expression (DE) results, a process that is labor-intensive and prone to inconsistencies or biases. In the context of marker identification, the package *MarkerPen* (Qiu *et al.* 2021) made some progress toward automated marker identification. However, it is semi-supervised and relies on predefined marker lists, thereby limiting their applicability to new datasets.

Although some scRNA-seq methods can technically be applied to labeled bulk RNA-seq data, due to the small sample sizes in bulk RNA-seq data, machine learning-based approaches are generally unsuitable. While for DE-based scRNA-seq methods, studies indicate they generally do not perform as well as DE-based methods specifically designed for bulk RNA-seq data (Squair *et al.* 2021, Heumos *et al.* 2023), underscoring the need for more tailored approaches. Moreover, many of the current tools’ workflows often fail to fully utilize the statistical information (e.g. *P* value or fold change) across multiple comparisons or datasets. These tools apply a direct intersection of the results, and therefore did not account for the effects of tissue-specific background expression, leading to nonspecific marker identification.

To address these challenges, we developed an R/Bioconductor package *mastR* (*M*arkers *A*utomated *S*creening *T*ool in *R*), offering integration of the following key features as a comprehensive framework: (i) an automated workflow that integrates statistical information across multiple DE comparisons and datasets, (ii) explicit consideration of tissue-specific background expression to enhance marker specificity.

*mastR* builds upon established DE analysis tools [*edgeR* (Robinson *et al.* 2010) and *limma* (Ritchie *et al.* 2015)] by implementing a rank-product-based scoring approach to integrate statistical information across multiple DE comparisons. This approach is particularly effective in scenarios where standard one-vs-all comparisons face limitations, such as when the target group shares high similarity with specific subgroup(s). While standard workflows might not identify markers that distinguish the target group from dissimilar groups due to the dominance of the similar subgroup in the analysis, *mastR*'s rank-product scoring method assigns balanced weights across all group comparisons. This design choice helps mitigate bias introduced by group size differences and enables the identification of markers that might be overlooked in standard approaches. Furthermore, *mastR* incorporates tissue-specific background expression through a signal-to-noise ratio (SNR) metric implemented in its marker selection algorithm, enhancing the specificity and reliability of identified markers. Through validation on both simulated and public datasets, we demonstrate that *mastR* achieves high accuracy and computational efficiency while maintaining robustness across diverse experimental contexts. These features make *mastR* valuable for both research applications and clinical marker identification where tissue context and complex group relationships need to be considered.

## 2 Methods

**Figure 1.**
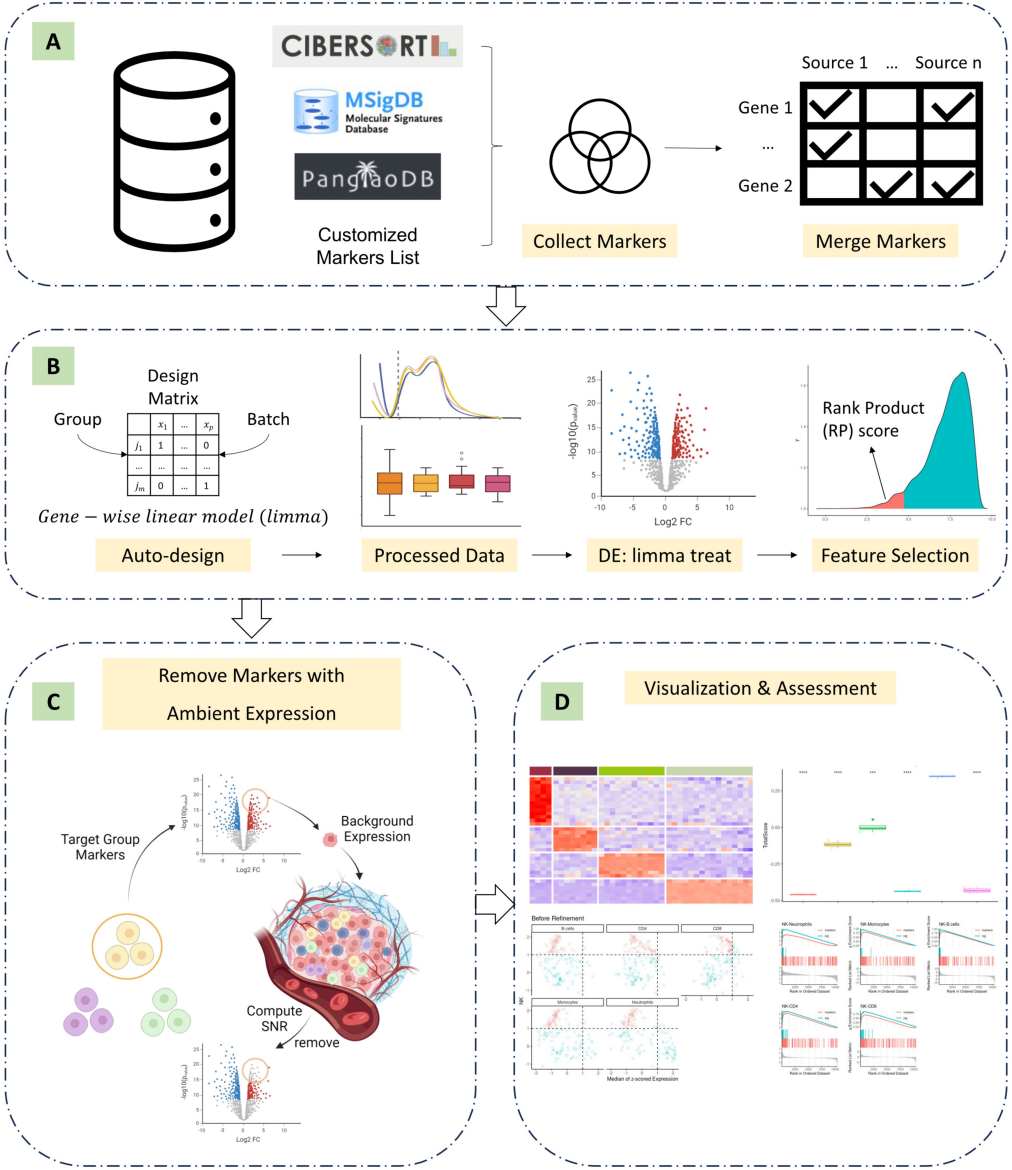
Schematic of the *mastR* workflow. The workflow of *mastR* can be divided into four main sections: (A) build markers pool; (B) identify signature markers; (C) refine signature by filtering based on background expression and (D) visualize and access signature performance. The *mastR* workflow recommends integrating markers from multiple sources (e.g. PanglaoDB, MSigDB) to form an initial set of markers. *mastR* then generates a design matrix based on the given “Group” and “Batch” factors to be used during data processing and DE analysis. The data processing includes an *edgeR* data filtering and normalization pipeline, and a *limma-voom-treat* based linear modeling DE approach to compare the target group with all other groups. *mastR* then computes the marker’s RP score based on the ranked product across the DE comparisons and bootstrapped permutation null distribution for further feature selection across multiple comparisons. The selected features will be constrained by their intersection with the initial set of markers. *mastR* allows for filtering of genes based on the SNR with a background dataset to remove features with inherent expression in a specific context or disease. *mastR* then provides visualization functions to assess the performance of the signature.

*mastR’s* workflow involves four steps (Fig. 1): (i) build a markers pool; (ii) identify the signature of the target group; (iii) refine the signature by removing the background signal of the sample microenvironment; and (iv) visualizing the resulting signature.

### 2.1 Build a marker pool

The *mastR* pipeline begins by generating a pool of candidate markers. This pool can be compiled either using the functions in *mastR* or by custom curation and selection of marker genes from databases or publications. For the former, the R/Bioconductor package *mastR* allows extraction of marker genes specific to immune cell types, relevant pathways, and/or gene sets from existing data sources, which can be retrieved via *get_lm_sig/get_panglao_sig/get_gsc_sig* functions in *mastR*. This includes leukocyte gene signature matrices from CIBERSORT [LM7 (Tosolini *et al.* 2017) and LM22 (Newman *et al.* 2015), immune cell signature matrices defining 7 and 22 immune cell types, respectively], PanglaoDB (scRNA-seq experiments from mouse and human that includes marker genes for 25 different immune cell types.) (Franzen *et al.* 2019), and MSigDB (Molecular Signatures Database, a collection of annotated gene sets) (Subramanian *et al.* 2005, Liberzon *et al.* 2015), respectively.

*mastR* provides *gsc_plot* function to help visualize the overlap of sets of markers. These sets of marker genes can be seamlessly merged as the original pool of markers using the *merge_markers* function in *mastR*, with all marker gene sources saved in the *longDescription* attribute. This merged pool will be used in subsequent analyses. When the markers pool is used in the downstream filtering step, all the marker genes in the pool will be preserved as these are determined to be of biological significance.

### 2.2 Identify signature of the target group

To identify group-specific signatures, *mastR* uses three main steps: (i) differential expression analysis (DEA), (ii) feature selection to select highly differentially expressed genes (DEGs) based on their rank-product score; and (iii) constraining selected genes within the markers pool (Fig. 1B).

Firstly, DEA is performed using *edgeR* (Robinson *et al.* 2010) and *limma* (Ritchie *et al.* 2015) workflow (i.e. filtering, normalizing, sample weighting and linear modeling). Given the “Group” and “Batch” factors in the data, *mastR* automatically generates the appropriate design matrix to be used during data filtering, normalization, and batch effect correction. Here, batch factor is used as the fixed effect in linear modeling as it was found that the use of batch-corrected data rarely improves the analysis of sparse data, whereas batch covariate modeling improves the analysis for substantial batch effects (Nguyen *et al.* 2023). *mastR* allows either raw counts or log-normalized data as input with different processing pipelines conducted on different types of input. Raw count data is filtered by the *filterByExpr* function in *edgeR*, normalized using the trimmed mean of M-values (TMM) method and analyzed using the *“limma voom with treat”* pipeline. For log-normalized data, genes are filtered by user-defined thresholds and *“limma trend with treat”* method is used. In most cases in this study, the log-fold-change (logFC) equal to 0 was used to perform DE analysis, with the only exception being when generating NK cell signature using DICE [Database of Immune Cell Expression, Expression quantitative trait loci (eQTLs) and Epigenomics] project (Schmiedel *et al.* 2018) in which case a logFC of 1.5 was used.

Secondly, feature selection is conducted to select genes specific to the target group across multiple comparisons. The probability ${\mathrm{score}}_{g}$ is computed by comparing the rank product (RP) score ${RP}_{g}$ with permutated random score rp from bootstrap approach [Equations (1)–(3)]. The common DE genes from n−1 comparisons (where n is the total number of groups) are identified and ranked based on the given gene statistics (e.g. *P* value, adjusted *P*-value or log fold change) for each comparison. The ranks for each marker gene across all comparisons are log-transformed and summed, before a permutation test was applied to bootstrap the null distribution of the random RP. The resulting marker genes are ordered by ${\mathrm{score}}_{g}$ (with the smaller the values being more significant) and filtered by a selected threshold (default as 0.05).


(1) $${RP}_{g}=\sum_{i=1}^{n-1}\ln\left({\mathrm{rank}}_{g,i}\right)$$

where ${\mathrm{rank}}_{g,i}$ is the rank of gene g in ${i\mathrm{th}}^{}$ list,


(2) $$rp=\left\{\sum_{i=1}^{n-1}\ln\left(r{\mathrm{rank}}_{g,i}\right)\right\}$$

where ${\mathrm{rrank}}_{g,i}$ is the shuffled rank of gene g in ${i\mathrm{th}}^{}$ list,


(3) $${\mathrm{score}}_{g}=P\left({RP}_{g}>rp\right)$$

The computation can be summarized as below:

1. Rank the gene statistics in increasing order (decreasing order of ‖logFC‖ when statistics is logFC) ⇒ ${\mathrm{rank}}_{g,i}$: rank of *g*th gene under *i*th comparison;
2. Sum log-rank for each gene across comparisons as ${RP}_{g}$: RP of *g*th gene;
3. Independently permute statistics value within each comparison relative to gene ID, repeat step (1)–(2) ⇒ ${rp}_{g}^{\left(k\right)}$: random RP of gth gene;
4. Repeat step (3) K times, form reference null distribution with ${rp}_{g}^{\left(k\right)}(k=1,2,\ldots ,K)$;
5. Determine the probability associated with each gene ⇒ ${\mathrm{score}}_{g}$.

In some marker identification studies, the presence of two or more closely related groups in the data pose challenges for the identified markers to be effective in distinguishable these groups (Burel *et al.* 2022). To accommodate this situation, threshold filtering based on RP can be omitted for the target comparison(s) in question by setting parameters “keep.top” and “keep.group,” allowing for a greater number of DEGs in the targeted comparison(s).

Thirdly, the identified marker genes are limited to those in the markers pool (i.e. common genes are retained) as the resulting signature. This refinement approach enhances both the discriminative power and the precision of the resulting signature when there is prior knowledge. When the input involves multiple datasets, *mastR* aggregates the individual signature lists identified by each dataset using either a “Robust Rank Aggregation (RRA),” “union,” or “intersect.”

The aggregation method “RRA” detects marker genes that are consistently ranked higher than stochastically expected under the null hypothesis of uncorrelated inputs and assigns a significance score to each gene (Kolde *et al.* 2012). It is recommended for robust gene selection from large numbers of DEGs. The “union” method is recommended for small numbers of marker genes identified per dataset; and the “intersect” method, is best used in situations characterized by high levels of marker intersection.

*mastR* provides a series of step-by-step functions as well as an integrated wrapper function to implement the above analyses.

### 2.3 Refine signature by accounting for background expression

To avoid background microenvironment confounding effects, *mastR* can further refine the marker genes by filtering out genes with ubiquitous expression. *mastR* utilizes an approach which remove genes with low “SNR” based on Cohen’s d (Knapp 1990), which have limited discriminative power between the group of interest and the “background” or “environment” [Equations (4) and (5)]. Considering situations where the background and signal expressions do not originate from the same batch, and that re-normalizing the entire data is time-consuming, in order to make the sample microenvironment and signal data comparable between batches, the relative expression of the genes within the samples are used to compute SNRs, making “signal” and “noise” comparable across datasets.

For DE analysis, we assume genes are not differentially expressed (null hypothesis) and the gene expression within each sample should follow a normal distribution denoted as $X∼N(\mu ,\sigma^{2})$. The parameters, mean (μ) and standard deviation (σ), can be estimated through maximum likelihood estimation (MLE). The percentile (accumulated density) for each gene in each sample can then be obtained using the Gaussian cumulative distribution function (CDF) $F\left(x|\mu ,\sigma^{2}\right)$, and the SNR computed as outlined in Equation (5).


(4) $${\hat{x}}_{S}=F^{-1}\left(0.5|\mu_{S},{\sigma_{s}}^{2}\right),\;{\hat{x}}_{B}=F^{-1}\left(0.5|\mu_{B},{\sigma_{B}}^{2}\right)$$

(5) $$\mathrm{snr}=\frac{{\hat{x}}_{S}-{\hat{x}}_{B}}{\sigma_{B}}$$

where $F^{-1}$ is the inverse CDF function, ${\hat{x}}_{S}$ represents the 50th percentile (median) of a normal distribution fitted to the observed log-transformed gene expression in the signal dataset S, ${\hat{x}}_{B}$ represents the 50th percentile (median) of a normal distribution fitted to the observed log-transformed gene expression in the background dataset B, μ is the mean of the normal distribution, σ is the standard deviation of the normal distribution, snr is the SNR of each gene.

This crucial step removes the effect of sample purity for the identified signature markers. By excluding the marker genes with similar expression in the sample microenvironment, the SNR approach ensures only the marker genes with robust and specific expression patterns in the group of interest are retained, leading to a more refined and accurate signature marker list.

## 3 Results

In this study, we evaluated *mastR*’s performance on both simulated and biological datasets, with *mastR* exhibiting high accuracy and robustness (Supplementary Figs S1–S5 and Supplementary Tables S3 and S4). Briefly, a natural killer (NK) cell specific signature from DICE dataset was identified using *mastR* (Supplementary Fig. S1) and validated in an independent immune cell dataset (Supplementary Fig. S4), showing high specificity for NK cells. The resulting performance metric on the simulated data (Supplementary Table S3) suggest *mastR* is highly accurate, have low false discovery rates and computationally fast.

We then compared the performance of the *mastR*-derived NK cells signature with existing published NK signatures with *mastR* demonstrating comparable, if not better performance in identifying NK cells (Supplementary Fig. S6). Assessing the average expression of the unique markers for each signature across the cell types, *mastR* is able to identify novel and highly specific marker genes for NK cells (Supplementary Fig. S7).

While the focus of this study was to evaluate *mastR* for marker identification in bulk RNA-seq data, we also looked at the potential application of *mastR* for scRNA-seq datasets. Here we compared *mastR*’s performance with Seurat (Hao *et al.* 2021), one of the statistical packages designed for scRNA-seq data. Interestingly, *mastR* performed better than Seurat and requires significantly lower computation time (Supplementary Table S4).

Till this end, we have assessed *mastR* for both bulk and scRNA-seq data, however it can theoretically be applied to all multi-omics data. Moving forward, the aim for this work is to validate the performance of *mastR* using experimental data across diverse omics types to improve application and generalizability across a range of research contexts.

## Supplementary Material

btaf114_Supplementary_Data

## Data Availability

The source code for *mastR* is freely available from the Bioconductor website at https://bioconductor.org/packages/release/bioc/html/mastR.html. Datasets are freely available for download from the following public data repositories and URLs. DICE (Database of Immune Cell Expression, Expression quantitative trait loci (eQTLs) and Epigenomics) at https://dice-database.org; TCGA-COAD at https://portal.gdc.cancer.gov/projects/TCGA-COAD; CCLE (Cancer Cell Line Data Repository) at https://sites.broadinstitute.org/ccle; im_data_6 at https://www.ncbi.nlm.nih.gov/geo/query/acc.cgi and can be accessed with GSE60424; pbmc3k.final at https://github.com/satijalab/seurat-data; NK Crinier at https://www.ncbi.nlm.nih.gov/pmc/articles/PMC6269138/; NK Cursons at https://aacrjournals.org/cancerimmunolres/article/7/7/1162/469488/A-Gene-Signature-Predicting-Natural-Killer-Cell and NK Shembrey at https://www.ncbi.nlm.nih.gov/pmc/articles/PMC9853446.
