# Supplementary material for: mastR: an R package for automated identification of tissue-specific gene signatures in multi-group differential expression analysis
Source: Bioinformatics. 2025 Mar 17;41(3):btaf114. doi: 10.1093/bioinformatics/btaf114 (PMC11937977; doi:10.1093/bioinformatics/btaf114)
Supplement: btaf114_Supplementary_Data [file btaf114_supplementary_data.pdf]

Supplementary Materials

Contents

Materials and Data 2
Extended Results 4
***mastR* automatically identifies specific gene expression signatures** 4
Marker genes identified by mastR are accurate and reproducible 10
The mastR-derived gene expression signature performs as well if not better than manually curated signatures 12
The mastR-derived signature as a potential indicator of clinical outcomes . 14
Supplementary References 20

Materials and Data

In this study, we accessed publicly available data including all the samples from the DICE (Database of Immune Cell Expression, Expression quantitative trait loci (eQTLs) and Epigenomics) project (https://dice-database.org) (Schmiedel, et al., 2018), colorectal cancer (CRC) RNA-seq data from The Cancer Genome Atlas (TCGA) (Cancer Genome Atlas, 2012), CRC cell line RNA-seq (TPM normalized) data from the Cancer Cell Line Encyclopedia (CCLE) (https://sites.broadinstitute.org/ccle) (Barretina, et al., 2012), whole transcriptome RNA-seq data of 6 sorted immune cell types of healthy individuals from GSE60424 (depicted as *im_data_6*, (Linsley, et al., 2014)) and a scRNA-seq peripheral blood mononuclear cell (PBMC) data (*pbmc3k.final*) in the Bioconductor R package *SeuratData* (Lab, 2020).

For comparisons of signatures, the published curated natural killer (NK) cell signatures from Crinier et al. (Crinier, et al., 2018) (named as *NK Crinier*), Cursons et al. (Cursons, et al., 2019) (named as *NK Cursons*) and Shembrey et al. (Shembrey, et al., 2022) (named as *NK Shembrey*) were used. All publicly available datasets and signatures are listed in **Supplementary Table 1**.

mastR provides a suite of visualization functions including heatmaps, box plots, scatter plots, rank density plots, and gene set enrichment analysis (GSEA) plots to facilitate the interpretation of the results and enable the evaluation of signature performances across multiple datasets. Details of all visualization functions of mastR can be found in **Supplementary Table 1**.

Supplementary Table 1. Datasets used in this study.

Data	Source	Date Version	Reference
**DICE**	Database of Immune Cell Expression, Expression quantitative trait loci (eQTLs) and Epigenomics	February 2018	PMID: 30449622
**TCGA-COAD RNA-seq**	The UCSC Cancer Genomics Browser	March 2023	PMID: 22810696
**CCLE RNA-seq**	Cancer Cell Line Data Repository	May 2023	PMID: 22460905
**GSE60424**	Gene Expression Omnibus	January 2015	PMID: 25314013
**pbmc3k.final**	SeuratData	pbmc3k 3.1.4	Satija lab Github[#]
**NK Crinier**	Crinier et al.	November 2018	PMID: 30413361
**NK Cursons**	Cursons et al.	July 2019	PMID: 31088844
**NK Shembrey**	Shembrey et al.	January 2023	PMID: 36685584

[#]-https://github.com/satijalab/seurat-data

Supplementary Table 2. Visualization Descriptions.

Visualization Function	Purpose
**heatmaps**	Provide an intuitive depiction of the expression levels of individual signature genes across various groups.
**box plots**	Assist in evaluating the overall separability of signatures between different groups by representing the distribution of signature scores within each group.
**scatter plots**	Allow for the examination of group specificity for each gene and the co-expression patterns between groups, which aids in the further refinement of gene selection.
**rank density plots**	Visually show the overall expression level of the signature in different groups.
**gene set enrichment analysis (GSEA) plots**	Simplify the demonstration of how given signature(s) are enriched in the target group compared to other groups.

Extended Results

mastR automatically identifies specific gene expression signatures

We tested *mastR*'s ability to automatically identify a natural killer (NK) cell specific signature from DICE dataset and validate that in an independent immune cell dataset (im_data_6). First, *mastR* summarizes a collection of NK markers from LM7, LM22, and PanglaoDB, along with the collection of gene sets associated with the "NATURAL_KILLER" term in MSigDB into a "pool" of markers. Then using this "pool", *mastR* automatically generates a set of NK signature genes from 15 immune cell types in the DICE dataset (Schmiedel, et al., 2018). We then compared the expressions of the *mastR*-derived NK signature genes in well-known cell types from both the training and independent datasets, with **Supplementary Figure 1** showing that the derived NK signature shows consistent performance with high specificity to NK cells. We compared NK cells with stimulated CD8+ T cells and B cells as they are the most similar and dis-similar cells to NK cells respectively. The derived NK cell markers are a subset of the original markers and found to be highly expressed in the NK cells and not well expressed in other cell types (e.g. B cells, CD8+ naïve T cells, **Supplementary Figure 1A, Supplementary Figure 2**). The derived NK signature can clearly distinguish NK cells from the other cell types as shown by the higher rank scores obtained by scoring each sample in DICE using *singscore* (**Supplementary Figure 1B**). As can be seen in **Supplementary Figure 1B**, significantly higher NK scores were demonstrated in NK cells, very clearly differentiating them from other immune cell types (**Supplementary Figure 3**).

We then validated the signature on an independent dataset *im_data_6* (GSE60424 (Linsley, et al., 2014)), which is a sorted bulk RNA-seq data from the blood of healthy individuals (**Supplementary Figure 1C-F and Supplementary Figure 4**). Our derived NK signature has distinctly higher expression in NK cells compared with CD8 or B cells (**Supplementary Figure 1C)** consistent with the results on the DICE dataset. This high specificity for NK cells is further illustrated by the normalized median expression biplot of NK cells against other cell types where the signature genes are located in the NK high / (B cells or CD8+ T cells low) quadrant (top-left, red points), distinct from the heterogenous distribution of the original markers in the pool (black points) (**Supplementary Figure 1D).** The normalized ranked density barcode of the signature on the NK, CD8 and B cells (**Supplementary Figure 1E)** suggests an enrichment of our NK signature genes in NK cells but not the other cell types. This enrichment is reinforced via GSEA showing significant over-representation of the signature genes in NK subsets (**Supplementary Figure 4D,** $p < 0.01$). Finally, the ranked scores of the signature clearly show the effective identification of NK cells from the other cell types (**Supplementary Figure 1F)**. Taken together, these results suggest that *mastR* can automatically derive a cell type expression signature that is highly specific.

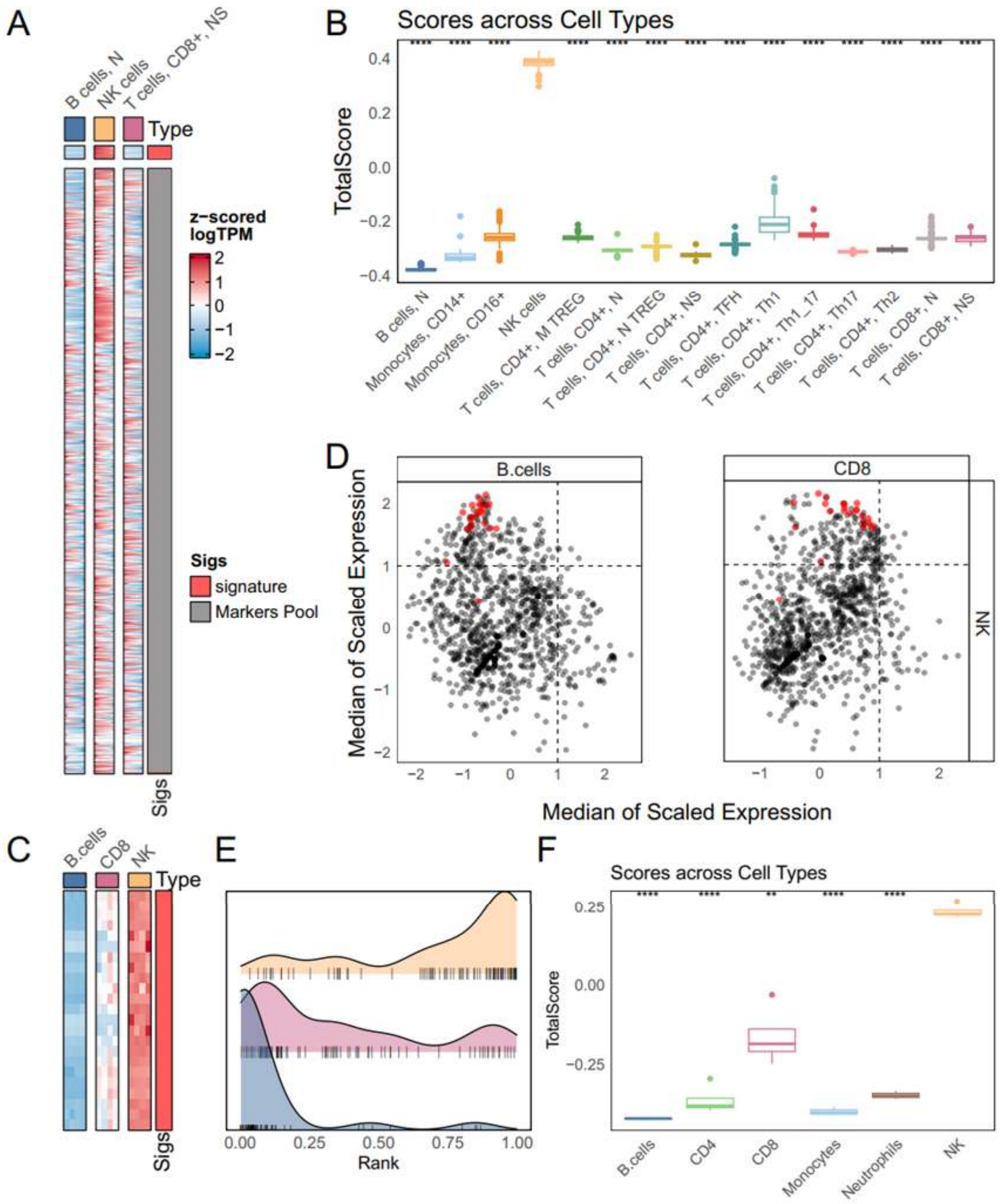

Supplementary Figure 1. The *mastR*-derived NK cell signature shows good performance on the immune datasets. Performance of *mastR*-derived NK signature on DICE (A-B) and on im_data_6 (C-F). **(A)** Heatmap of scaled log gene expression of all genes in the original markers pool with top section representing the 24 genes from the *mastR*-derived NK signature in DICE; **(B)** Boxplot of ranked scores (using *singscore*) of *mastR*-derived NK signature for the different cell types in DICE; **(C)** Heatmap of scaled log gene expression of *mastR*-derived NK signature in im_data_6; **(D)** Scatter plot of z-scored median gene expression in NK cells (y axis) against z-scored median gene expression in either B cells or CD8+ T cells (x axis) for all genes in markers pool, *mastR*-derived NK signature genes are highlighted in red, with the top-left quadrant represents regions of high NK-specificity; **(E)** Normalized rank density ridges plot of *mastR*-derived NK signature in NK cells (top), B cells (middle) and CD8+ T cells (bottom) respectively; **(F)** Boxplot of ranked scores (using *singscore*) of *mastR*-derived NK signature for the cell types in im_data_6. * M = memory; N = naïve; S = Stimuli in (A) and (B).

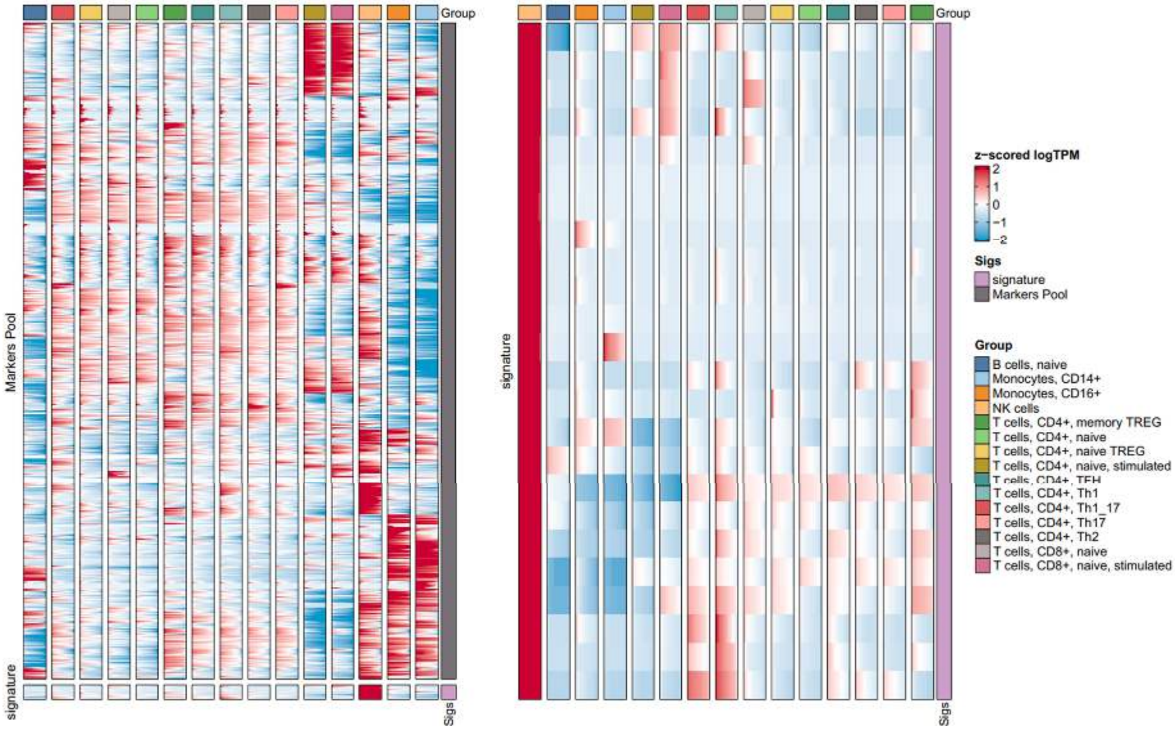

***Supplementary* Figure 2. *mastR*-derived NK signature is enriched in NK cells.** Heatmap of scaled gene expression (logTPM) of NK cell marker genes in DICE. (Left) Heatmap of the whole original set of markers across all cell types in DICE. (Right) Heatmap of the *mastR*-derived NK signature genes across all cell types in DICE.

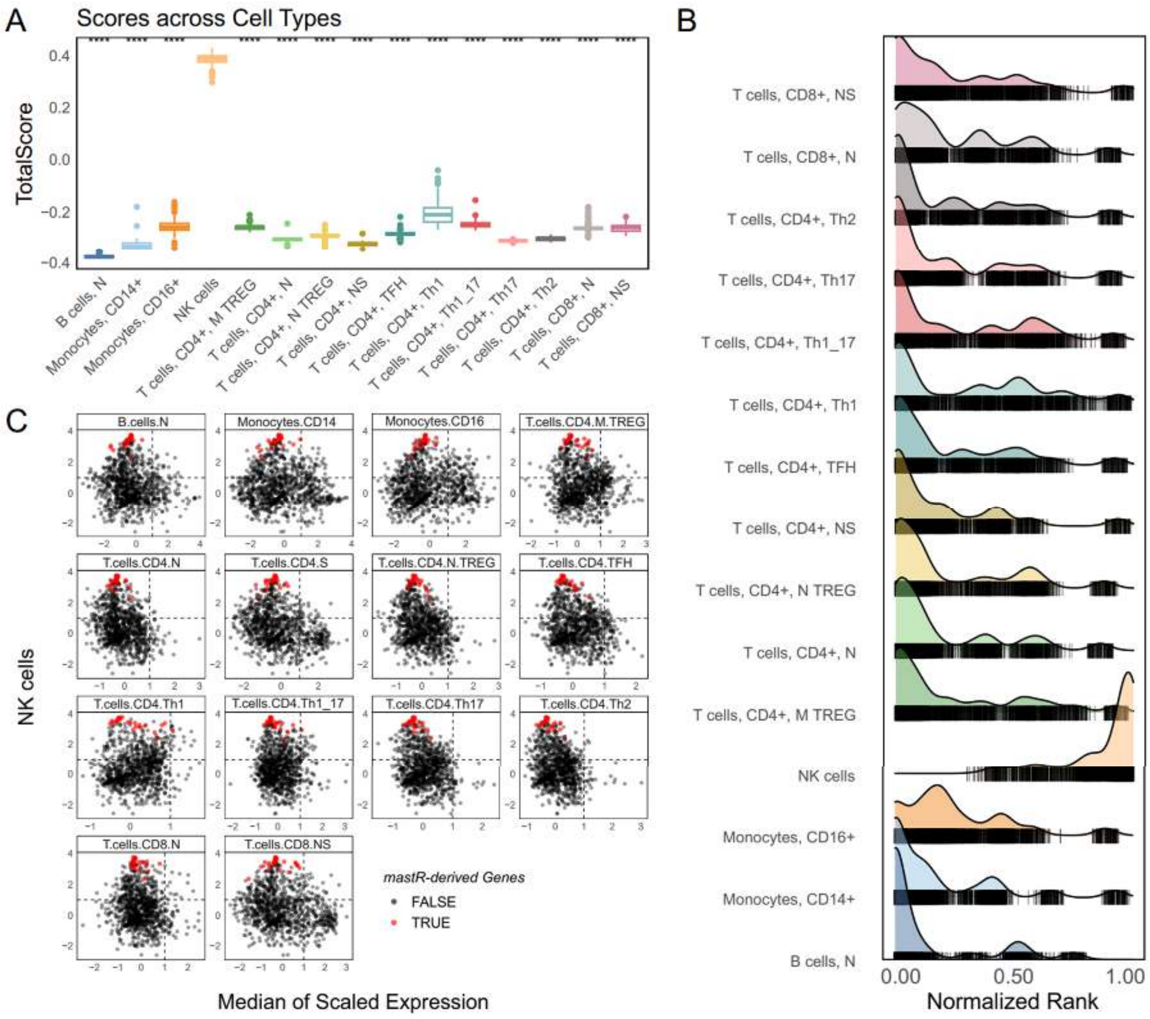

Supplementary Figure 3. The *mastR*-derived NK signature is highly specific to NK cells in DICE. Overall performance of NK signature in DICE dataset. **(A)** Boxplot of ranked scores (using *singscore*) based on derived NK signature across cell types in DICE; **(B)** Normalized rank density ridges plot of derived NK signature for each cell type; **(C)** Scatter plot of z-scored median gene expression in NK cells (y axis) against z-scored median gene expression in other cell types (x axis) for all the genes in the original set of markers. The *mastR*-derived NK signature genes are highlighted in red, top-left quadrant represents region of high NK-specificity. * M = memory; N = naïve; S = Stimuli in (A), (B) and (C).

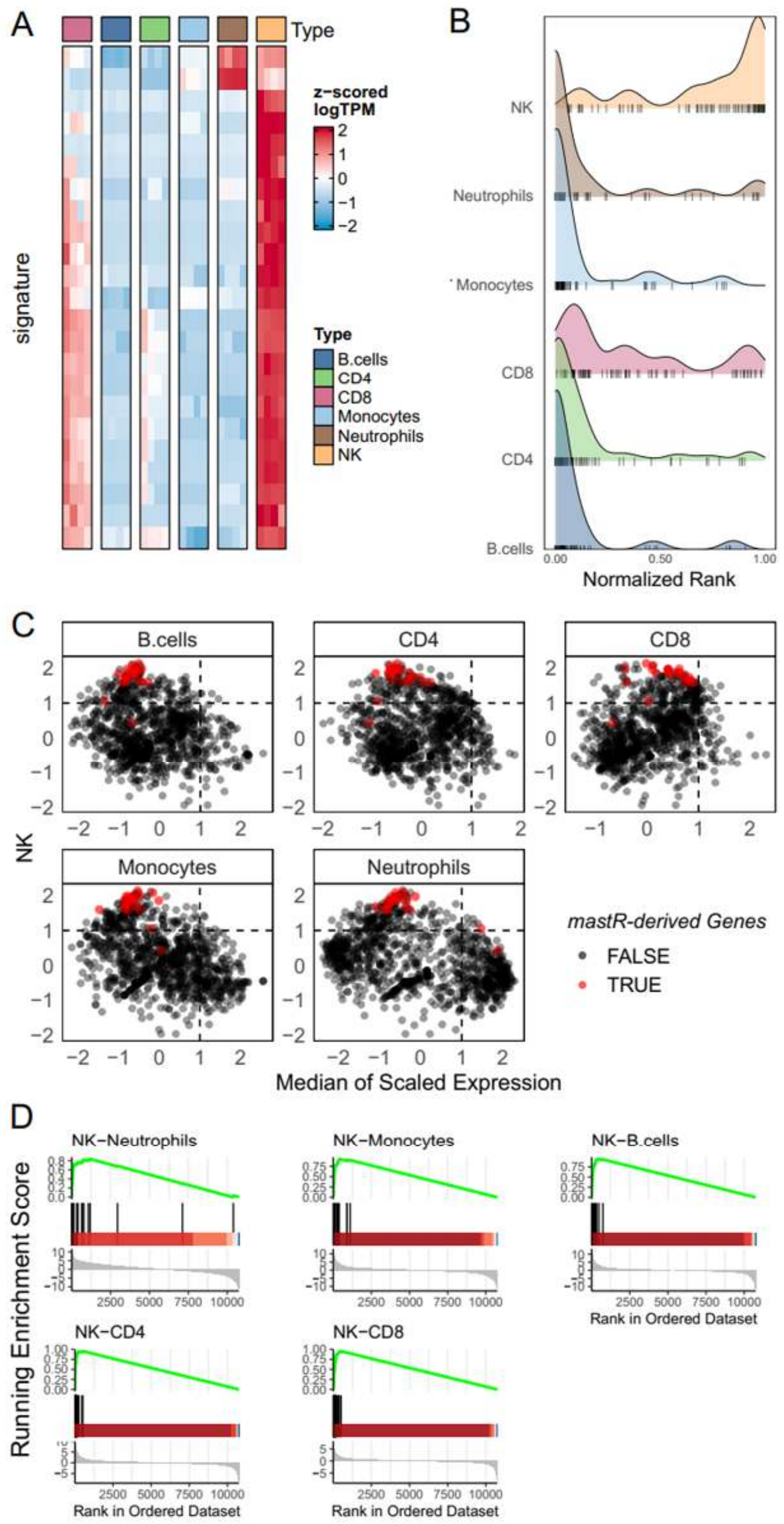

Supplementary Figure 4. The *mastR*-derived NK signature performs well when applied on the independent dataset *im_data_6*. Performance overview of derived NK signature in *im_data_6*. **(A)** Heatmap of scaled log gene expression of NK signature across cell types in *im_data_6*; **(B)** Normalized rank density ridges plot of NK signature for each cell type in *im_data_6*; **(C)** Scatter plot of z-scored median genes expression in NK cells (y axis) versus other cell types (x axis) in *im_data_6*, *mastR*-

derived NK signature genes are highlighted by red, left-top quadrant represents high NK-specificity; **(D)** Gene-set Enrichment Analysis (GSEA) results of *mastR*-derived NK signatures for the comparisons of NK vs other cell types in im_data_6 presented as running enrichment scores against barcode plots (all p < 0.01).

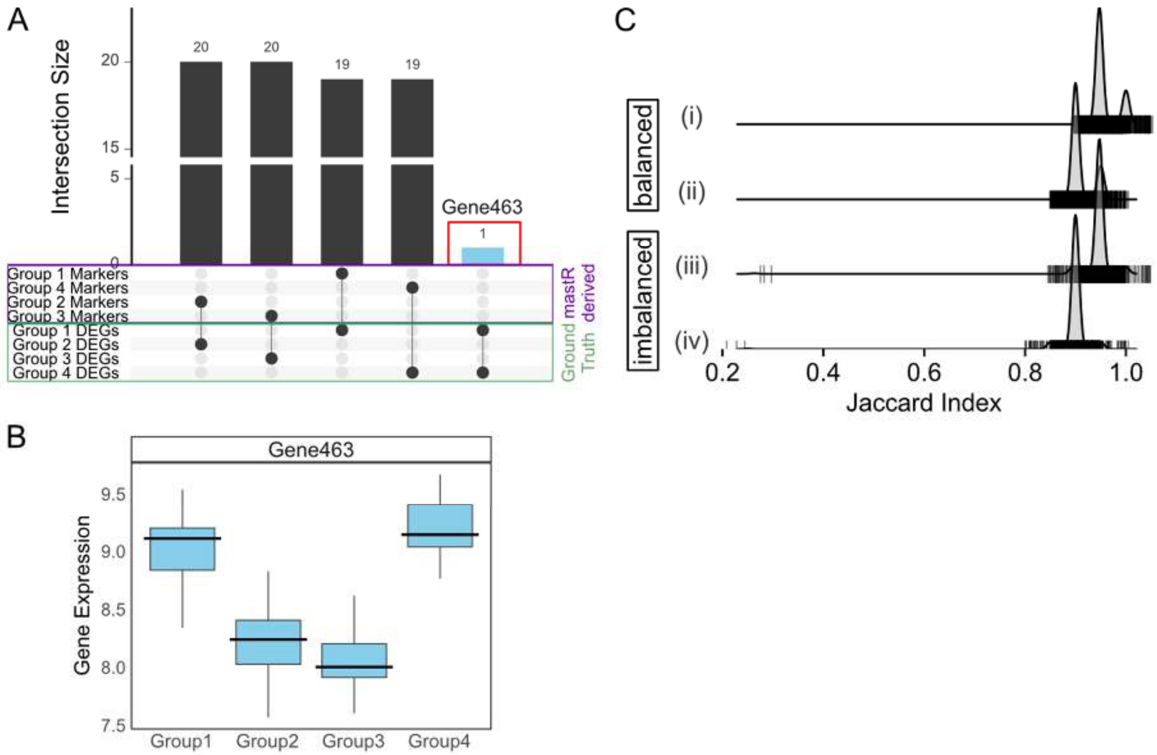

Supplementary Figure 5. The *mastR* is able to accurately and robustly identify DE markers. (A) Upset plot of *mastR*-derived group signatures ("Group 1-4 Markers") and simulated up-regulated DEGs ("Group 1-4 DEGs", the ground truth). Note *mastR* did not identify gene "Gene463" which is commonly DE in both Group 1 and 4 of the DEGs ground truth groups (in blue); **(B)** Boxplot of the expression (logCPM) for the simulated up-regulated DEG "Gene463" shared between Group 1 and 4; **(C)** Ridges plot of Jaccard index (JI) across 1,000 random samples of the simulated bulk RNA-seq data. Ridges plot of JI for balanced (top, 80% of each group sampled to obtain new signature) and im-balanced (bottom, 40%, 50%, 70%, and 80% of groups 1 to 4 sampled respectively) sampling strategy on simulated bulk RNA-seq data. The curves (i) & (iii) represent JI between the *mastR*-derived new signature (from the sub-samples) and the derived original signature (from the whole dataset), and (ii) & (iv) represent JI between the *mastR*-derived new signature (from sub-samples) and the simulated DEGs (ground truth) (ii & iv).

Marker genes identified by mastR are accurate and reproducible

We assessed the reliability and robustness of *mastR* approach using a simulation study. Bulk RNA-seq data (100 samples across 4 groups, 1000 genes at 2% DE rate with balanced composition) was simulated as per described in the Methods section. We first identified the signature for each group using *mastR* on the whole simulated dataset, finding all identified signature genes overlap exactly with the ground truth (i.e. simulated differentially expressed genes (DEGs), **Supplementary Figure 5A**. However, in the simulated ground truth data, there is one shared DEG between Group 1 and Group 4 ("Gene463", colored blue in **Supplementary Figure 5A**) which is not identified and not amongst the *mastR*-derived signatures. This gene has similar high expression in both Group 1 and Group 4 as compared to the rest of the groups (**Supplementary Figure 5B)**. This suggests *mastR* only identifies highly specific genes across the groups, with no false positive markers identified.

We then test the reproducibility of the *mastR* by applying it onto sub-sampled datasets with the same group compositions. For each subset, we randomly selected 80% of the samples from each group to generate new group signatures and compared this with the signature derived earlier using the whole dataset using the Jaccard index (JI, Jaccard similarity coefficient). The resulting JI distribution in **Supplementary Figure 5C** (for balanced data) indicates a high similarity with JI > 0.85 (sampling of 1,000), suggesting that *mastR* can stably reproduce the signatures regardless of the size of the dataset. Similarly, we evaluated the sensitivity of *mastR* to varying group sizes using an imbalanced sampling strategy where the sampling probabilities for each group are 0.4, 0.5, 0.7, and 0.8 (groups 1 to 4 respectively). Similar results are obtained as shown in **Supplementary Figure 5C** (for imbalanced data), with similarity JI of > 0.85 (medians of JI, sampling of 1,000). This implies that *mastR* can accurately identify group-specific marker genes regardless of dataset sizes or difference in group composition in a robust manner.

The stability and performance of *mastR* was further evaluated as per the 1000 simulations as described by computing the precision, recall, F1 score and false positives metrics by comparing the derived signatures with the simulated ground truth. Results indicate that *mastR* achieves excellent performance with a F1 score of 0.97 ± 0.04, 100% precision, a recall of 0.95 ± 0.06 and low false positives rate of 0.01 ± 0.07 when comparing *mastR*-derived signatures with all simulated DEGs (**Supplementary Table 3**, "all DEGs"). A smaller recall was noted compared to the F1 score, suggesting some "DEGs" that may not be identified by *mastR*. These specific "DEGs" were further assessed and found to be highly specific for more than just one group, as was the case for "Gene463" (**Supplementary Figure 5B)**. Thus, we refined the comparison to only compare the derived signatures with only the simulated DEGs that's unique to each group. In this case, the performance improved as expected with an F1 score of 0.99 ± 0.03 and a recall of 0.99 ± 0.05 (**Supplementary Table 3**). The results demonstrate *mastR* can accurately identify marker genes with high specificity,

particularly for those unique marker genes to each group in the data.

Supplementary Table 3. Performance on simulated bulk RNA-seq data.

metric	mean	sd	DEGs
F1 Score	0.97	(0.04)	All
	0.99	(0.03)	Unique
Precision	1.00	(0)	All
	1.00	(0)	Unique
Recall	0.95	(0.06)	All
	0.99	(0.05)	Unique
False Positives	0.01	(0.07)	
Running Time	2.91	(0.39)	

Although *mastR* was originally designed for bulk RNA-seq data, we further assessed its applicability on scRNA-seq data simulated using the *splatter* (Zappia, et al., 2017) package as described (3000 cells across 4 groups, each 1000 genes at 2% DE rate with balanced composition). The single cells were aggregated (pseudo-bulked) and performance evaluated against commonly used scRNA-seq package *Seurat* (Hao, et al., 2021) for 1,000 simulations. Results as shown in **Suppplementary Table** , suggest that *mastR* achieves better performance than *Seurat* on simulated scRNA-seq across all the performance metrics, with higher accuracy and lower false positives. Importantly, it should be noted that *mastR* is significantly more computationally efficient (~ 1.29 seconds on average) compared with *Seurat* (~ 60 seconds on average) which is important for real life applications. Therefore, *mastR* can handle large-scale single cell datasets with complex scenarios at a lower computational cost, showing a significant speed advantage and high accuracy.

Suppplementary Table 4. Performance of mastR and Seurat on simulated scRNA-seq data.

metric	mastR		Seurat		DEGs
	mean	sd	mean	sd	
F1 score	0.94	(0.02)	0.83	(0.05)	All
	0.88	(0.02)	0.77	(0.04)	Unique
Precision	1.00	(0.01)	0.79	(0.05)	All
	0.88	(0.03)	0.70	(0.04)	Unique
Recall	0.89	(0.03)	0.88	(0.04)	All
	0.87	(0.03)	0.87	(0.04)	Unique
False Positives	0.00	(0)	2.31	(1.4)	
Running Time	1.29	(0.14)	59.76	(5.41)	

**The total running time was measured for signature identification and screening using mastR on a standard computer resource (including pseudo-bulk processing time).*

The mastR-derived gene expression signature performs as well if not better than manually curated signatures

We further explored the performance of our automatically generated NK signature (*NK mastR*) compared to the published manually curated signatures, using an independent dataset *pbmc3k.final* (Lab, 2020). Three published NK cell signatures (Crinier, et al., 2018; Cursons, et al., 2019; Shembrey, et al., 2022) were collated for this study, namely from Crinier *et al (NK Crinier*), Cursons *et al* (*NK Cursons*) and Shembrey *et al* (*NK Shembrey*). All 4 signatures can distinguish NK cells from all other cell types when comparing normalized rank scores, with CD8+ T cells most closely resembling NK cells (**Supplementary Figure 6**). However, for *NK Crinier* and *NK Cursons*, the majority of CD8+ T cells exhibit a higher score than the lowest score of the NK cells (arrows in **Supplementary Figure 6A**), indicating a higher degree of similarity between these 2 signatures. This is also shown in the mean differences of the ranked scores between NK cells and CD8+ T cells, where both *NK Crinier* and *NK Cursons* have the smallest differences (< 0.40). This observation is further validated using the area under the precision-recall curve (AUPRC) in **Supplementary Figure 6B**, where only *NK mastR* and *NK Shembrey* achieved an AUPRC of > 0.90. This suggests that the automatically generated NK signature is performing as well if not better than manually curated signatures in distinguishing between cell types.

We then access the gene signatures at the individual gene level where it is noted that most of the signature genes have high expression in NK cells while less so for CD8 T cells (**Supplementary Figure 6C)**. However, it should be noted that both *NK Cursons* and *NK Crinier* also have relatively higher expression in CD8 T cells compared to the other signatures. On the other hand, a significant number of *NK Shembrey* signature genes also have low expression in both NK and CD8 T cells. By comparing the overlapping genes between the signatures in **Supplementary Figure 6D**, each signature has a set of unique genes (in blue) with only *KLRF1* (an essential gene expressed in most NK cells) common amongst all 4 signatures. We assessed the performance of these unique genes by comparing their average expression within NK cells or CD8+ T cells (**Supplementary Figure 6E)**. Most of the unique genes in our NK signature is highly specific to NK cells while exhibiting low expression and proportions in CD8+ T cells, while other signatures are either specific to NK and CD8+ T cells (*NK Cursons* and *NK Crinier*) or have many unique genes non-specific to NK cells (*NK Shembrey*). These results are also found to hold up when comparing across all cell types (**Supplementary Figure 7**), confirming the ability of *mastR* to identify novel and highly specific markers.

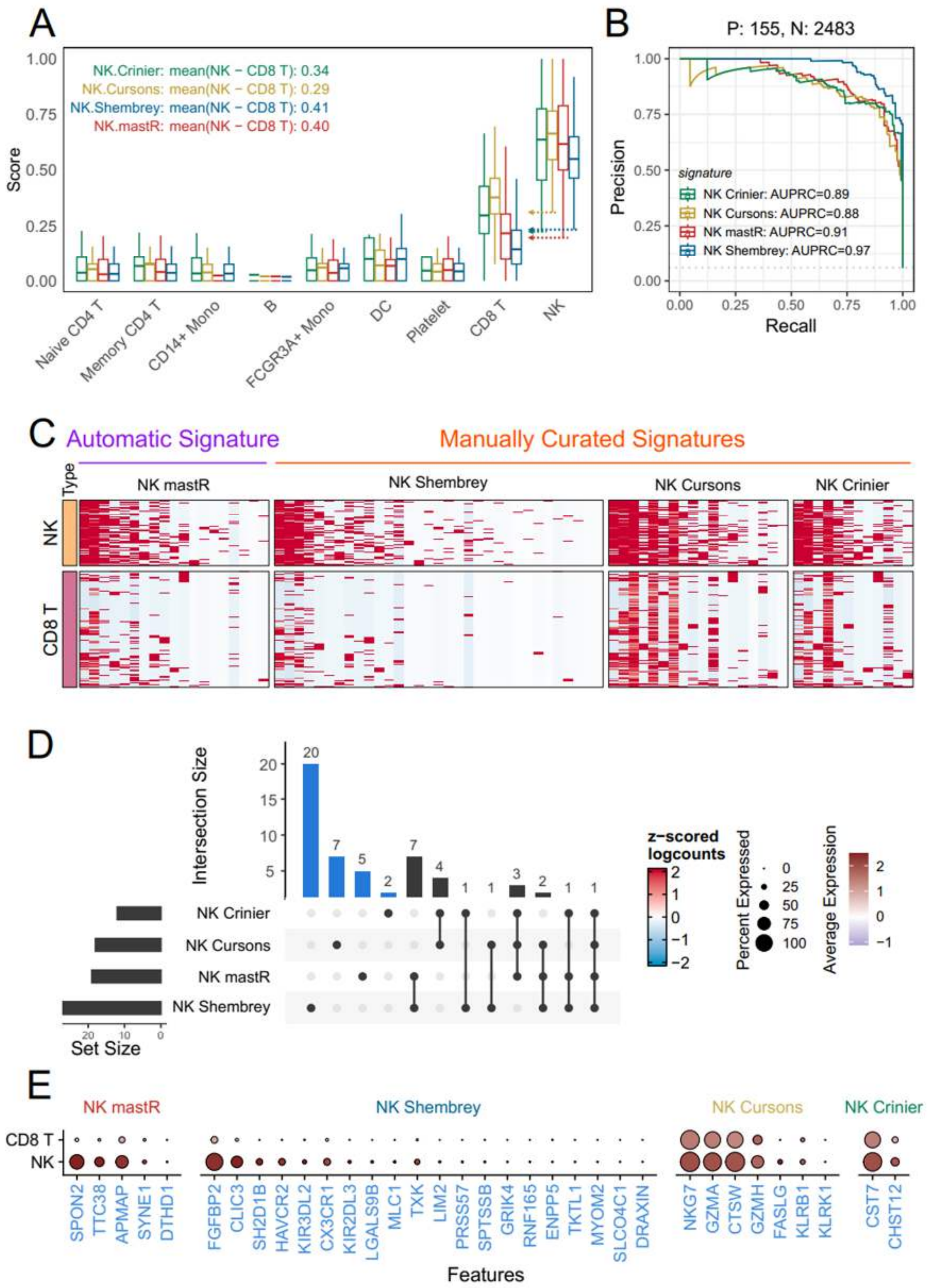

Supplementary Figure 6. The *mastR*-derived NK signature performance as well as published curated signatures. Comparisons of signatures performance using independent scRNA-seq data (pbmc3k.final). **(A)** Boxplot of scaled ranked scores (using *singscore*) on the 4 NK signatures of interest with the mean differences in ranked scores between NK and CD8 T cells calculated and shown (inset). The minimum scores of NK cells for each signature are shown as a colored arrow for each boxplot; **(B)**

Precision-recall curve (PRC) of the NK signatures with the individual area under curve (AUC) computed; **(C)** Heatmap of scaled log gene expression of the 4 NK signatures for both CD8+ T cells and NK cells; **(D)** Overlap between the 4 NK signatures. Blue bars indicate numbers of unique genes for each signature; **(E)** Dot plot of the average gene expression of the unique signature genes in each signature (blue bars in *D*) for CD8+ T cells and NK cells, color represents average expression and size represents expressed percentage. * Note that only genes in pbmc3k.final dataset are shown and used in subsequent analysis.

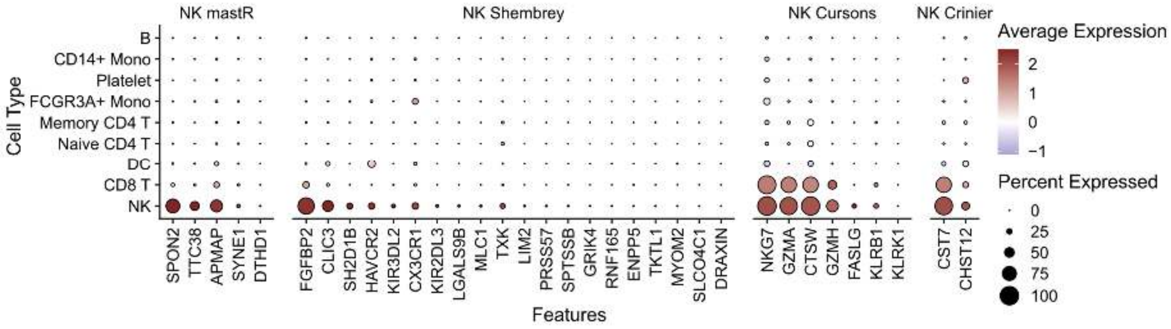

Supplementary Figure 7. Dot plot of average expression of the unique genes for each NK signature across the cell types in pbmc.3k.final. Color represents average expression, size represents percent of expressed cells.

The mastR-derived signature as a potential indicator of clinical outcomes

We further assess how our derived NK signature can be applied to clinical applications. Here we apply our NK signature to the TCGA-COAD dataset (**Supplementary Figure 8-10**) to assess how well it can delineate clinical outcomes. In this analysis, the colorectal-associated cell lines data from CCLE (Barretina, et al., 2012) was used as the reference data to remove colon specific signature genes (i.e. highly expressed with SNR < 1 in CRC-related cell lines). Each sample was then rank scored and the subsequent NK scores were compared across different clinical indicators. The results suggest that NK signature has no significant correlation with prior malignancy status or gender (**Supplementary Figure 8**). However, significantly different NK signature ranked scores were observed across various clinical indicators, indicating different extents of NK cell infiltration under different conditions. In particular, amongst the consensus molecular subtypes (CMS), CMS 1 subtype has a significantly higher NK score than the other subtypes (**Figure 4A**). This is consistent with the findings from the study by Guinney and colleagues (Guinney, *et al*., 2015) where it was suggested that CMS 1 subtype tumors have a strong immune response and higher NK cell activity. Our results also indicate a significantly elevated NK ranked score in microsatellite instability (MSI) high samples compared to others (**Supplementary Figure 11**). This potentially suggests increased NK cell infiltrations in MSI high tumors which agrees with a previous finding by Lanuza *et al*. (Lanuza, et al., 2022).

Taking the clinical indicators showing significant differences in NK scores, survival analyses were performed where we found that overall survival (OS) is significantly associated with clinical disease stage, M (metastatic) stage and prior treatment status (**Supplementary Figure 9**), while progression-free interval (PFI) is significantly associated with clinical stage, M stage and CMS groupings (**Supplementary Figure 10**). These results suggest that patients at a more advanced disease stage or M stage (**Supplementary Figure 11C**, top & middle), show poorer outcomes in terms of both OS and PFI, and both conditions are associated with a low NK score. Interestingly, CMS survival analysis presents a different trend for PFI, where CMS1 (which had the highest NK ranked scores amongst the CMS subtypes) (**Supplementary Figure 11A**) shows a poor PFI similar with that of CMS4 (**Supplementary Figure 11C**, bottom). This was also observed in the study by Guinney *et al* (Guinney, et al., 2015) which also concluded that both CMS1 and CMS4 presents the worst relapse-free outcomes. This suggests that CMS1 is a heterogenous cancer subtype where survival outcomes are associated with multiple factors and not solely due to NK cell infiltration. Thus, to investigate the contribution of NK signature ranked score to CRC survival outcomes, the samples were categorized into 3 groups based on the 30th and 90th percentile of NK ranked scores: depicted as low, medium and high respectively (**Supplementary Figure 11D**, top). Subsequent survival analysis for PFI (**Supplementary Figure 11D**, middle & bottom) shows a significant association between NK ranked scores and the patients' PFI, where a higher NK score is associated with lower risk. Taken together, these results suggest that *mastR*-derived NK signature can potentially be applied to clinical data as an indicator of clinical outcomes.

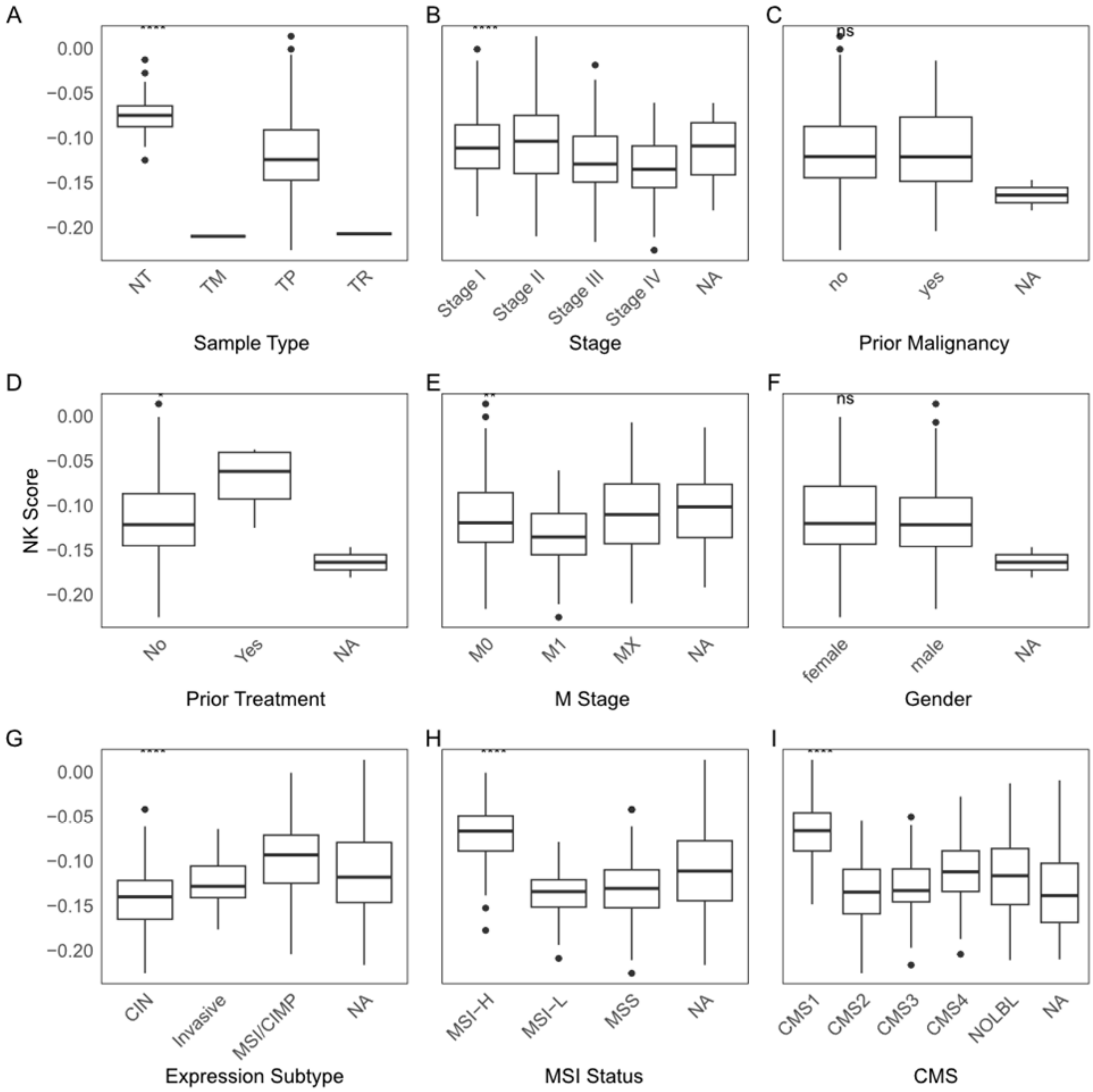

Supplementary Figure 8. NK signature score shows significant differences between clinical indicators. Boxplot of the ranked scores of derived NK signature for different clinical conditions in TCGA-COAD dataset. NK ranked scores were plot based on (A) tissue types, (B) cancer stages, (C) malignancy status, (D) treatment status, (E) metastatic (M) stages, (F) gender, (G) expression subtypes, (H) microsatellite instability (MSI) status and (I) consensus molecular subtypes (CMS). * p-value $\leqslant$ 0.05; ** p-value $\leqslant$ 0.01, *** p-value $\leqslant$ 0.001, **** p-value $\leqslant$ 0.0001, ns p-value > 0.05.

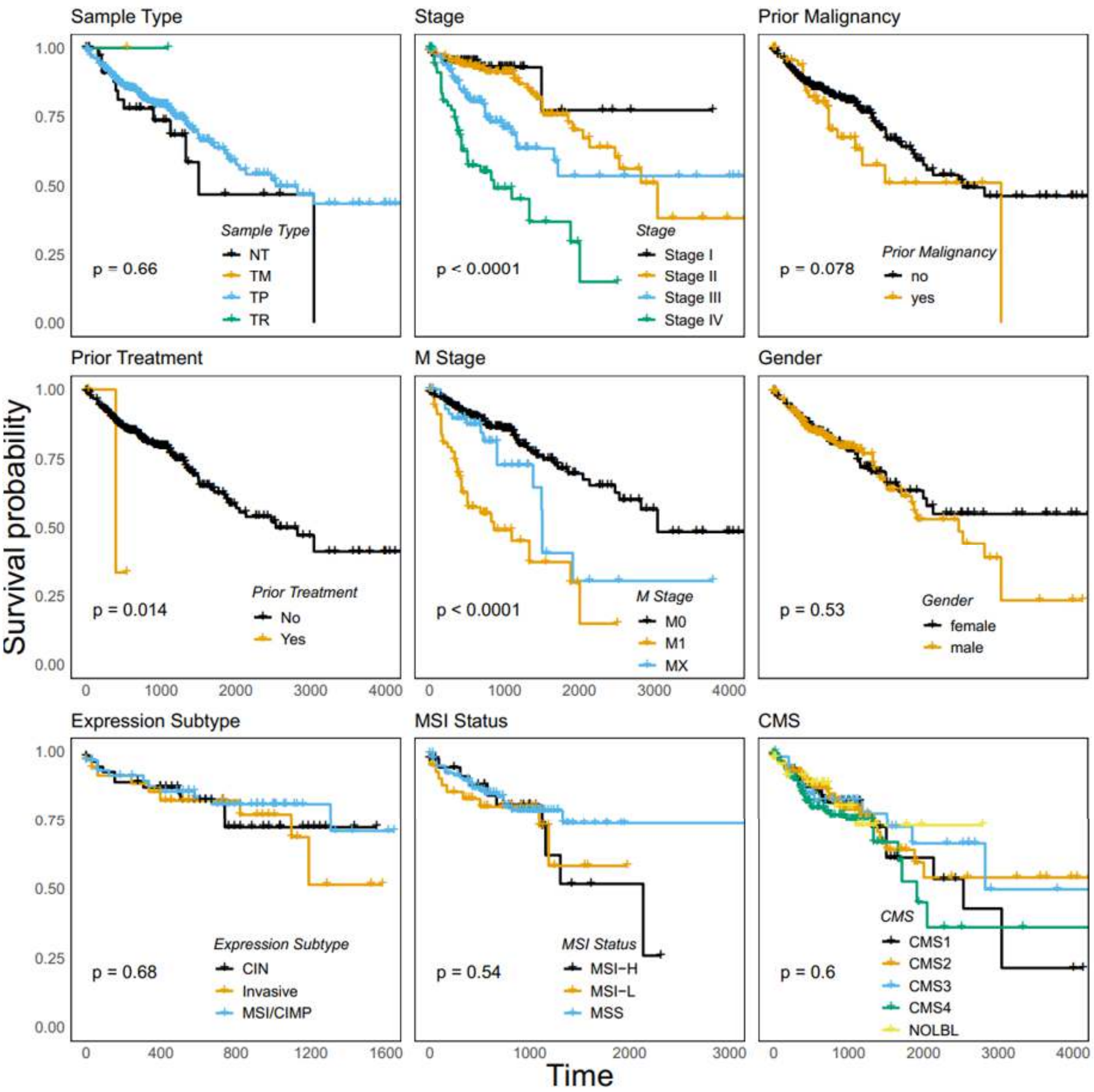

Supplementary Figure 9. Survival analysis for overall survival (OS) across clinical indicators in TCGA-COAD dataset. Survival analysis was conducted using packages *survival* and *survminer* using default parameters, a total of 522 samples from 458 patients were analyzed, with log-rank test p value shown.

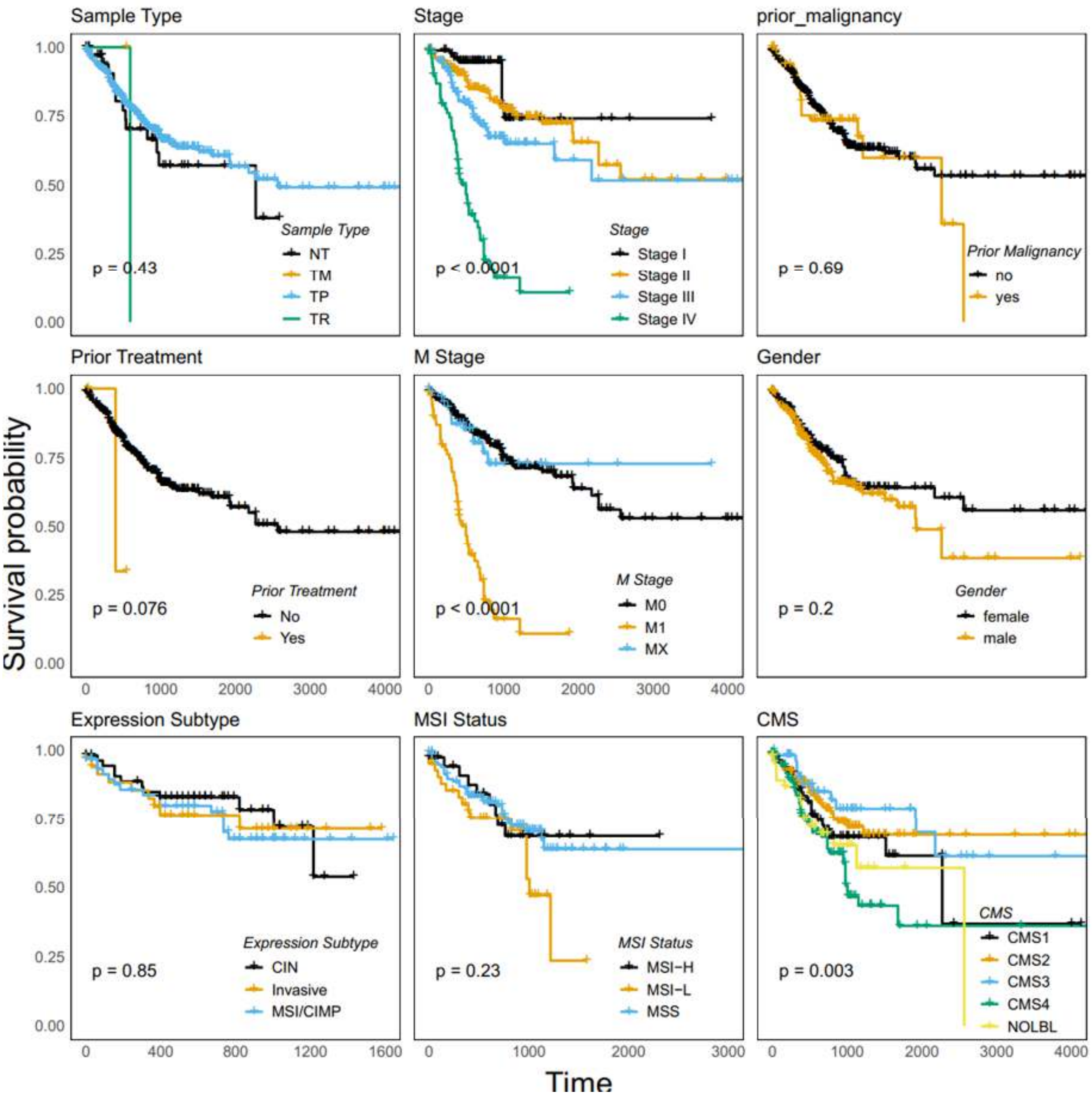

Supplementary Figure 10. Survival analysis for progression-free interval (PFI) across clinical indicators in TCGA-COAD dataset. Survival analysis was conducted using packages *survival* and *survminer* using default parameters, a total of 522 samples from 458 patients were analyzed, with log-rank test p value shown.

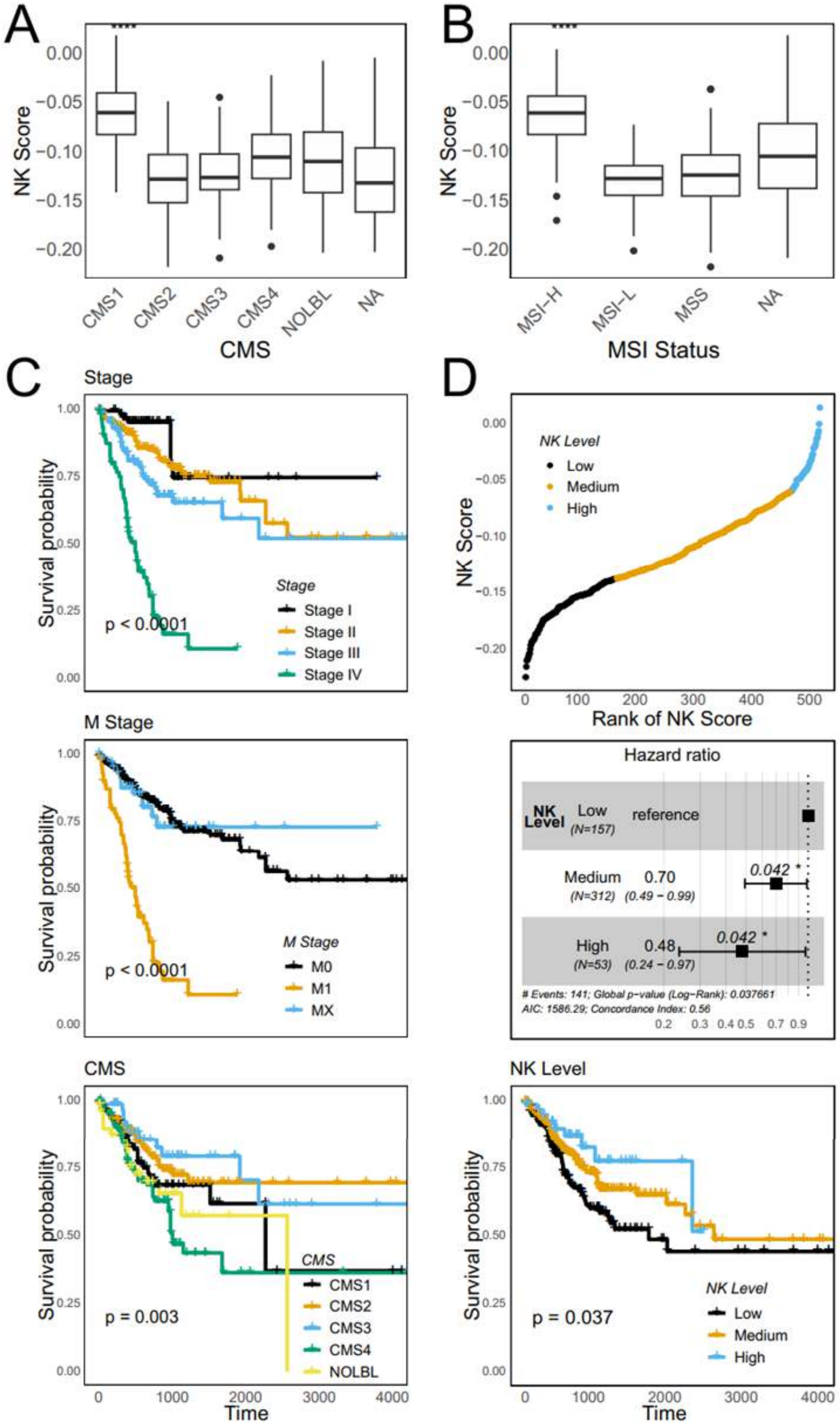

Supplementary Figure 11. Application of NK signature on TCGA-COAD dataset. (A) Boxplot of NK ranked scores (using *singscore*) across (A) consensus molecular subtypes (CMS) and **(B)** microsatellite instability (MSI) groups, with significance calculated by t test; **(C)** Survival analysis of progression-free interval (PFI) across clinical stages (top), metastatic (M) stages (middle) and CMS groups (bottom) with log-rank test p value; **(D)** Samples are categorized into 3 groups based on the 30th and 90th percentile

of NK ranked scores with samples ordered by NK ranked scores (top); forest plot of Cox proportional hazards model of PFI on NK score groups (middle); and survival analysis of PFI on NK score groups with log-rank test p value (bottom). Survival analyses are conducted using packages *survival* and *survminer* with default parameters on 522 samples from 458 patients.

Supplementary References

Barretina, J., *et al.* The Cancer Cell Line Encyclopedia enables predictive modelling of anticancer drug sensitivity. *Nature* 2012;483(7391):603-607.

Cancer Genome Atlas, N. Comprehensive molecular characterization of human colon and rectal cancer. *Nature* 2012;487(7407):330-337.

Crinier, A., *et al.* High-Dimensional Single-Cell Analysis Identifies Organ-Specific Signatures and Conserved NK Cell Subsets in Humans and Mice. *Immunity* 2018;49(5):971-986 e975.

Cursons, J., *et al.* A Gene Signature Predicting Natural Killer Cell Infiltration and Improved Survival in Melanoma Patients. *Cancer Immunol Res* 2019;7(7):1162-1174.

Guinney, J., *et al.* The consensus molecular subtypes of colorectal cancer. *Nat Med* 2015;21(11):1350-1356.

Hao, Y., *et al.* Integrated analysis of multimodal single-cell data. *Cell* 2021;184(13):3573-3587 e3529.

Lab, S. pbmc3k.SeuratData: 3k PBMCs from 10X Genomics. In.; 2020.

Lanuza, P.M., *et al.* Adoptive NK Cell Transfer as a Treatment in Colorectal Cancer Patients: Analyses of Tumour Cell Determinants Correlating With Efficacy

and. *Frontiers in Immunology* 2022;13.

Linsley, P.S., *et al.* Copy Number Loss of the Interferon Gene Cluster in Melanomas Is Linked to Reduced T Cell Infiltrate and Poor Patient Prognosis. *Plos One* 2014;9(10).

Schmiedel, B.J., *et al.* Impact of Genetic Polymorphisms on Human Immune Cell Gene Expression. *Cell* 2018;175(6):1701-1715 e1716.

Shembrey, C., Foroutan, M. and Hollande, F. A new natural killer cell-specific gene signature predicting recurrence in colorectal cancer patients. *Front Immunol* 2022;13:1011247.

Zappia, L., Phipson, B. and Oshlack, A. Splatter: simulation of single-cell RNA sequencing data. *Genome Biol* 2017;18(1):174.
